# Anti-adipogenic and anti-obesity activities of purpurin in 3T3-L1 preadipocyte cells and in mice fed a high-fat diet

**DOI:** 10.1186/s12906-019-2756-5

**Published:** 2019-12-11

**Authors:** Woo Nam, Seok Hyun Nam, Sung Phil Kim, Carol Levin, Mendel Friedman

**Affiliations:** 1 Department of Biological Science, Ajou University, Suwon, 16499 Republic of Korea; 2 Research Institute of Basic Science, Ajou University, Suwon, 16499 Republic of Korea; 3 STR Biotech Co., Ltd., Chuncheon, 24232 Republic of Korea; 4 Western Regional Research Center, Agricultural Research Service, U.S. Department of Agriculture, Albany, California, 94710 USA

**Keywords:** Adipogenesis, Anthraquinone, Antioxidant, Cell differentiation, Lipogenic diet, Madder, Mitochondria, Obesity, *Rubia cordifolia*

## Abstract

**Background:**

The body responds to overnutrition by converting stem cells to adipocytes. In vitro and in vivo studies have shown polyphenols and other natural compounds to be anti-adipogenic, presumably due in part to their antioxidant properties. Purpurin is a highly antioxidative anthraquinone and previous studies on anthraquinones have reported numerous biological activities in cells and animals. Anthraquinones have also been used to stimulate osteoblast differentiation, an inversely-related process to that of adipocyte differentiation. We propose that due to its high antioxidative properties, purpurin administration might attenuate adipogenesis in cells and in mice.

**Methods:**

Our study will test the effect purpurin has on adipogenesis using both in vitro and in vivo models. The in vitro model consists of tracking with various biomarkers, the differentiation of pre-adipocyte to adipocytes in cell culture. The compound will then be tested in mice fed a high-fat diet. Murine 3T3-L1 preadipocyte cells were stimulated to differentiate in the presence or absence of purpurin. The following cellular parameters were measured: intracellular reactive oxygen species (ROS), membrane potential of the mitochondria, ATP production, activation of AMPK (adenosine 5′-monophosphate-activated protein kinase), insulin-induced lipid accumulation, triglyceride accumulation, and expression of PPARγ (peroxisome proliferator activated receptor-γ) and C/EBPα (CCAAT enhancer binding protein α). In vivo, mice were fed high fat diets supplemented with various levels of purpurin. Data collected from the animals included anthropometric data, glucose tolerance test results, and postmortem plasma glucose, lipid levels, and organ examinations.

**Results:**

The administration of purpurin at 50 and 100 μM in 3T3-L1 cells, and at 40 and 80 mg/kg in mice proved to be a sensitive range: the lower concentrations affected several measured parameters, whereas at the higher doses purpurin consistently mitigated biomarkers associated with adipogenesis, and weight gain in mice. Purpurin appears to be an effective antiadipogenic compound.

**Conclusion:**

The anthraquinone purpurin has potent in vitro anti-adipogenic effects in cells and in vivo anti-obesity effects in mice consuming a high-fat diet. Differentiation of 3T3-L1 cells was dose-dependently inhibited by purpurin, apparently by AMPK activation. Mice on a high-fat diet experienced a dose-dependent reduction in induced weight gain of up to 55%.

## Background

**Fig. 1:**
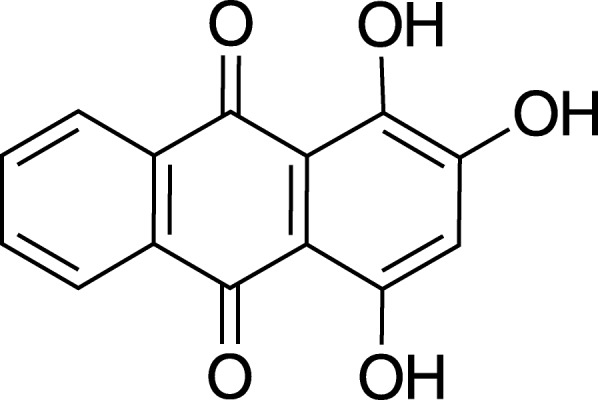
Structure of purpurin

Anthraquinones are aromatic compounds with a 9,10-dioxoanthracene core substituted in the two benzene rings with phenolic OH and aliphatic groups. Anthraquinones have numerous reported biological activities, reviewed in Li and Jiang [1]. Purpurin, 1,2,4-trihydroxy anthraquinone (Fig. 1), is an alizarin-type anthraquinone with particularly high anti-oxidative activity [1, 2]. Purpurin, and the related anthraquinone alizarin, are present as glycosides in the roots of the madder plant, the common name for both *Rubia tinctorum* L. and *Rubia cordifolia* [3]. These anthraquinones are responsible for the ancient natural pigments extracted from the madder plant used to dye textiles and color paints [4]. *Rubia cordifolia* contains primarily the purpurin glycoside, whereas *Rubia tinctorum* L. contains primarily the alizarin glycoside [3]. While not eaten for nourishment, the madder root has been used as a food colorant [5], and in traditional and conventional medicines to treat various aliments [6–8].

Purified purpurin has been the subject of various inquiries as to its biologic activity. It appears to have anti-angiogenesis activity [9], anti-mutagenic activity [10], anti-carcinogenic [11] and adjuvant activity [12], anti-inflammatory activity [13], anti-fungal activity [14], and anti-bacterial activity [15]. Purpurin’s high antioxidant capacity may be responsible for many of its bioactivities [2]. Malik, et al. [16] reviewed the use of flavonoids and anthraquinones as oxidase inhibitors for medicinal applications. Oxidative stress is not only linked to the diseases associated with obesity and metabolic syndrome, but also with the hypertrophy and hyperplasia of adipocytes [17].

A decrease in the volume of adipose tissue can be achieved by various means via: negative energy balance; inhibition of proliferation of cells; increase in apoptosis of cells; inhibition of differentiation of pre-adipocytes to adipocytes; inhibition of cellular lipid accumulation; and stimulation of lipolysis [18]. Polyphenols and other similarly antioxidative natural compounds have demonstrated anti-obesity effects by one or more of these processes [19, 20]. Of the anthraquinones, rhein [21] and emodin [22] have been shown to have anti-adipogenic activity. Interestingly, in the above experiment with emodin, Yang, Yuan, Hao and Lu [22] found a concurrent increase in osteogenesis, supporting a possible link between the differentiation of the two cells lines, osteoblasts and adipocytes, which are both derived from mesenchymal stem cells. It is poignant that several evidence-based studies have tested anthraquinones against osteoporosis stemming from its traditional use to treat bone ailments in China, reviewed in An, et al. [23]. To our knowledge, purpurin has not been evaluated for any of these activities.

In vitro study of adipogenesis is most often accomplished using murine fibroblast 3T3-L1 cells. This cell line, first developed in 1974 [24], can be stimulated to differentiate into adipocyte-like cells under prescribed conditions. Relevant to this study, the model has been used extensively to study effects of natural substances on adipose cells [25]. In addition to microscopic examination, signaling molecules, transcription factors, and kinases can be monitored to assess adipogenesis [18].

The current study here has two major objectives: to apply purpurin to an in vitro adipogenesis assay, measuring the common biomarkers associated with the process, and to confirm the expected reduction in weight gain and expression of obesity-associated biomarkers in a mouse assay in which mice were fed a high-fat diet supplemented with purpurin.

## Methods

### Materials

Dulbecco’s modified Eagle’s medium (DMEM), phosphate-buffered saline (PBS), bovine calf serum (BCS), fetal bovine serum (FBS), and other miscellaneous cell culture reagents were purchased from Hyclone Laboratories (Logan, UT, USA). Purpurin, 3-isobutyl-1-methylxanthine (IBMX, aka MIX), dexamethasone (DEX), insulin, 2,7-dichlorofluorescein diacetate (DCF-DA), JC-1, and 3-(4,5-dimethylthiazol-2-yl)-2,5-diphenyl tetrazolium bromide (MTT) were the products of Sigma-Aldrich (St. Louis, MO, USA). All reagents of analytical grade were purchased from Sigma-Aldrich and used without further purification and modification. MDI (MIX-DEX-Insulin)-differentiation medium consisted of DMEM containing 10% FBS, 0.5 mM IBMX, 1 μM dexamethasone, 1 μg/mL insulin, 100 U/mL penicillin, and 100 μg/mL streptomycin. 3T3-L1 murine pre-adipocyte cells purchased from the American Type Tissue Culture Collection (Manassas, VA, USA).

### Cytotoxicity of purpurin

Cell viability was assessed using the MTT staining assay following the method of Mosmann [26]. This method has previously been applied to 3T3-L1 cells [27, 28]. Briefly, cells were treated with serial concentrations of purpurin (0, 1, 10, 50, 100, 250 μM) for 24 h or 48 h at 37 °C in humidified air with 5% CO_2_. After treatments, the cells were stained by adding MTT, and the absorbance read at 570 and 655 nm.

### In vitro adipogenesis

#### Cell culture and induction of differentiation

**Fig. 2:**
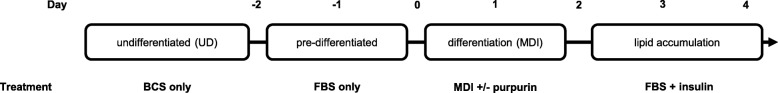
Scheme of 3T3-L1 pre-adipocyte differentiation and purpurin treatment

Cells were induced to differentiate according to methods used previously [27, 28]. Briefly, 3T3-L1 murine pre-adipocyte cells were stimulated to differentiate by culturing in MDI medium in the presence and absence of purpurin for 2 days. At this point cells were tested for early-stage differentiation biomarkers. The cells were then allowed to fully differentiate by culturing in DMEM for eight additional days [29]. Figure 2 shows the cell-culturing protocol.

#### Assays of purpurin-treated cells in early-stage differentiation

As described above, 3T3-L1 cells were cultured for 48 h in 6-well plates with nontoxic concentrations 50 μM and 100 μM purpurin in the presence of MDI medium to induce differentiation. Cells were detached using a cell scraper and transferred to 1.5 mL brown tube before being microcentrifuged at 13,000 rpm for 5 min. The pellet was assayed as described below.

##### Intracellular reactive oxygen species (ROS) and mitochondrial membrane potential (MMP)

The intracellular ROS level was determined [30] simultaneously to MMP using fluorescence labeling with DCF-DA and JC-1, respectively. Following the addition of 20 μM DCF-DA or 5 μM JC-1 to the pellet, incubation continued for 30 min in the dark at 37 °C, before washing twice with PBS. Labeled cells were resuspended in 200 μL PBS and transferred to a 96-well black plate. The fluorescence was monitored using a fluorescence plate reader (model SpectraMax Gemini XS, Molecular Device, Sunnyvale, CA, USA). The excitation wavelength and emission wavelength, respectively, were as follows: 485 nm and 535 nm for DCF-DA, 485 nm and 530 nm for JC-1 green fluorescence, and 550 nm and 600 nm for JC-1 red fluorescence. MMP was calculated by dividing the red fluorescence by the green fluorescence. The measured fluorescence is expressed as a percent of the control group.

##### Adenosine triphosphate (ATP) assay

The intracellular ATP level was determined using StayBrite™ Highly Stable Luciferase/Luciferin Reagent (BioVision Inc., Milpitas, CA, USA). Cells were lysed with ATP lysis buffer (50 mM Tris-Cl, 150 mM NaCl, 10 mM MgCl_2_, and 1 mM EDTA, pH 7.5) added to the pellet. After centrifugation, 200 μL of supernatant was mixed with 10 μL luciferase/luciferin reagent and 1 μL DTT. The luminescence was measured using TD-20/20^n^ luminometer (Turner Biosystems, Sunnyvale, CA). The measured luminescence was quantified using a standard curve and normalized by the amount of DNA in the cells.

##### Western blot analysis for p-AMPK and AMPK

AMPK, p-AMPK, and β-actin were determined by Western Blot according to our previous method [28]. Briefly, cells were lysed with RIPA to release cell proteins, which were quantitated according to the Bradford method using a Bio-Rad (Hercules, CA) protein assay kit. The cell extracts (30 μg) were then separated on 10% SDS-polyacrylamide gels and electrophoretically transferred onto nitrocellulose membrane (Millipore, Billerica, MA, USA). The membrane was blocked then probed with anti-phospho-AMPK rabbit polyclonal antibody and anti-AMPK rabbit polyclonal antibody (Cell Signaling Technology, Danvers, CA, USA); and anti-β-actin monoclonal antibody (Millipore, Billerica, MA, USA). The secondary antibody reaction was with HRP (horseradish peroxidase)-conjugated anti-IgG antibody under the same conditions. Blots were developed using the ECL detection kit (Pierce, Rockford, IL, USA), and quantified using a gel documentation system (Vilber Solo S, Vilber Lourmat, Paris, France).

#### Assays of purpurin-treated cells in late-stage differentiation

After the initial 48 h of MDI treatment, cells were cultured in DMEM containing 10% FBS, 1 μg/mL insulin, 100 U/mL penicillin, and 100 μg/mL streptomycin (Fig. 2), with the media changed every 2 days. At day 6 post-MDI treatment, cells were tested for mid-stage transcription factors and proteins.

##### Western blot analysis for PPARγ, C/EBPα, and β-actin

PPARγ, C/EBPα, and β-actin were analyzed by Western blot by the same procedure as above on cells in mid-stage differentiation. The antibodies used were anti-C/EBPα rabbit polyclonal antibody (Cell Signaling Technology), anti-PPARγ monoclonal antibody (Cell Signaling Technology), and anti-β-actin monoclonal antibody (Millipore, Billerica, MA, USA).

#### Assays of purpurin-treated differentiated cells

Cells were fully differentiated at day 8 post-MDI treatment. Cellular fat and protein accumulation were assessed by direct assay and by microscopic examination.

##### Oil red O staining

Morphological changes were assessed by the method of Oil Red O staining as done previously [28]. Briefly, cells were fixed with 4% paraformaldehyde in PBS at 4 °C for 24 h, washed and dried, then stained with Oil Red O for 1 h in the dark. Visualization was by light microscopy (model D50, Olympus, Tokyo, Japan) and photography [31]. Lipid accumulation was quantified by solubilizing the stained lipid droplets with 2-propanol and reading the absorbance at 520 nm.

##### Triglycerides

Triglycerides were determined according to previous methods [28]. Briefly, cells were harvested with the use of accutase (Sigma-Aldrich). Total lipids were extracted using a commercial kit (Sigma-Aldrich). Triglyceride content was measured using a commercial enzymatic assay kit (Wako, Osaka, Japan) according to the manufacturer’s instructions. Triglyceride content was reported relative to cellular protein, which was determined according to the Bradford method using a Bio-Rad Protein assay kit [32].

### In vivo feeding studies

Feeding studies in mice were done according to a previous method [28]. Pathogen-free male C57BL/6 mice, 6–8 weeks old, were obtained from Orient Bio Inc. (Seoul, Republic of Korea). The mice were housed in groups of 5 in a stainless-steel cage under a 12 h light/dark cycle with a temperature range of 20–22 °C and relative humidity of 50 ± 10%. Mice were fed the pelletized normal commercial chow diet (Cat. No.5 L79, Orient Bio.) and tap water ad libitum for 1 week after arrival for acclimation. After acclimation, mice were arbitrarily divided into the following four groups (*n* = 10): standard diet (SD), high-fat diet only (HFD), high-fat diet with 40 mg/kg purpurin (HFD 40), and high-fat diet with 80 mg/kg purpurin (HFD 80). Standard diet consisted of 14% fat, 21% protein, and 65% carbohydrate. High-fat diet (D12492, Research Diet, New Brunswick, NJ, USA) consisted of 60% fat, 20% protein, and 20% carbohydrate; 6% of the fat came from soybean oil and 54% from lard. Purpurin was administrated via the diet. The amount added was recalculated weekly based on mouse weights and food intake. A sufficient amount of food was fed to allow the mice to eat freely. Food efficiency ratio (FER) was calculated using the following formula; FER = body weight gain (g)/total food intake (g).

#### Glucose tolerance test

After 10 weeks, feed was withheld for more than 20 h before performing a glucose tolerance test. Fasting mice were orally administered 200 μL of glucose at a dose of 1 g/kg body weight. Blood was collected from the tail vein, and the blood glucose level was determined using an AccuChek Active Kit (Roche Diagnostics, Mannheim, Germany) at 0, 15, 30, 60, and 120 min after glucose challenge [33].

#### Analysis of biochemical indices in serum and histology of the adipose and liver tissues

At the end of the experimental period, the mice were weighed, and then sacrificed by CO_2_ inhalation. After blood collection by cardiac puncture, the subcutaneous epididymal white adipose tissues and the liver were removed from the mice and weighed immediately. Blood samples rested at 4 °C for 30 min to induce blood clotting, and were then microcentrifuged at 3000 g for 30 min at 4 °C. The resultant serum (supernatant) was stored at − 70 °C until use. Serum triglyceride levels were quantitated following the method described above. Serum levels of total cholesterol were analyzed using a commercial kit (Asan Pharmaceuticals, Seoul, Korea) according to the manufacturer’s instructions. Serum enzyme glutamate oxaloacetate transaminase/glutamate pyruvate transaminase (GOT/GPT) concentrations were determined using a colorimetric assay kit (Asan Pharmaceuticals) following the manufacturer’s protocol. For histological analysis, the tissues were fixed with 4% paraformaldehyde in phosphate buffer (0.5 M, pH 7.4), rinsed with water, dehydrated with ethanol, and embedded in paraffin. Then, the tissues were cut to a thickness of 4 μm and mounted onto charged glass slides. The sections were dewaxed using xylene and ethanol, stained with hematoxylin and eosin Y (H & E), and assessed using light microscopy for the quantitation of adipocyte size and detection of fat accumulation lesions in the liver.

### Statistical analysis

Results are expressed as the mean ± SD of three independent experiments. Significant differences between means were determined by the one-way ANOVA test followed by Duncan’s multiple range test using the Statistical Analysis Software package SAS (Cary, NC, USA). *p* < 0.05 is regarded as significant.

## Results

### Purpurin-induced effects in 3T3-L1 cells

#### Cytotoxicity of purpurin

**Fig. 3:**
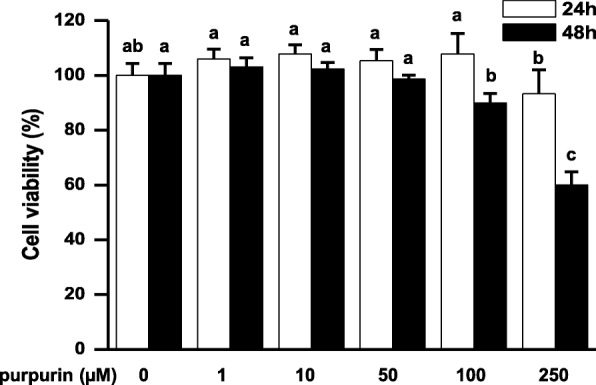
Cytotoxicity of purpurin on the murine 3T3-L1 pre-adipocyte cell line. Cells were cultured in the presence of purpurin (1, 10, 50, 100, and 250 μM) for 24 and 48 h. After treatment, cell survival was measured by the MTT assay. Data are expressed as the mean ± SD of triplicate experiments. Bars sharing a common letter are not significantly different at *p* < 0.05

To check for potential toxicity of purpurin on 3T3-L1 cells, viability after exposure to a series of concentrations of purpurin, and for 2 time periods, was determined using the MTT assay. The results showed (Fig. 3) that at the highest concentration tested, 250 μM, viability decreased only slightly and non-significantly at 24 h, but by about 40% by 48 h. The 100 μM concentration showed no effect at 24 h, and only about a 10% decrease at 48 h. This concentration (100 μM) was chosen as the maximum used for all further studies.

#### Biomarkers in early-stage differentiation

**Fig. 4:**
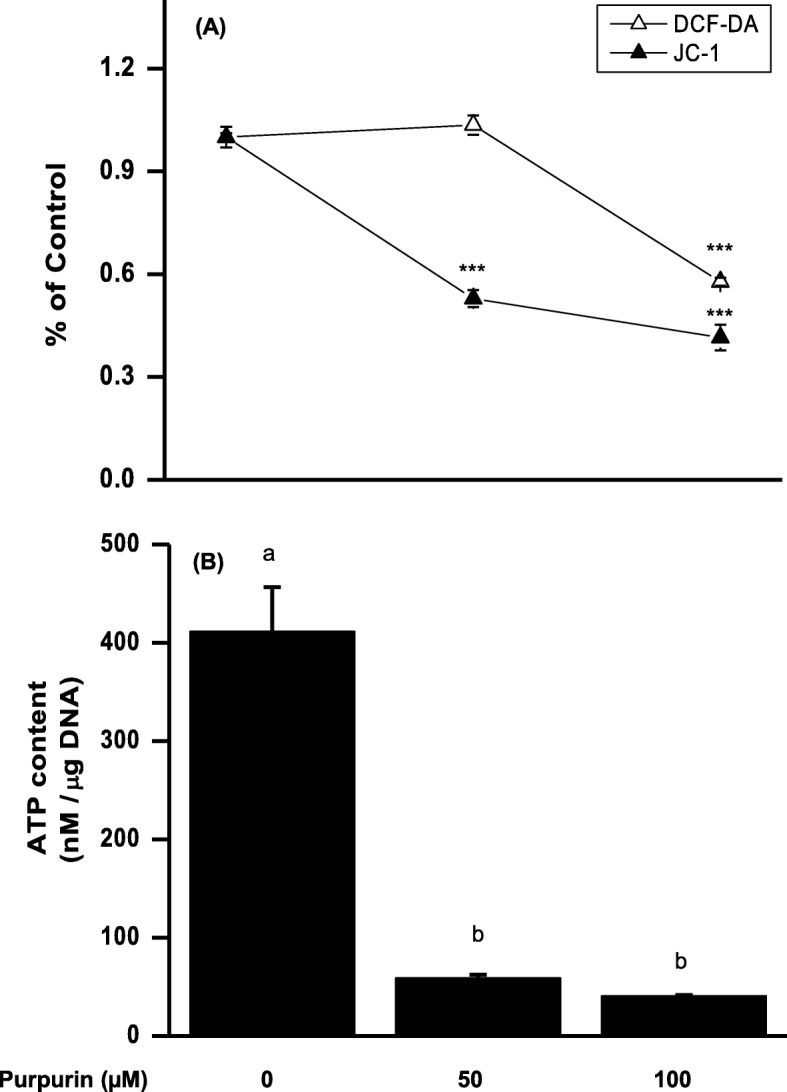
Effects of purpurin on intracellular ROS levels, mitochondrial membrane potential, and intracellular ATP levels in 3T3-L1 cells treated with MDI for 48 h: **a** cells were labeled with DCF-DA for cellular ROS detection, and JC-1 for the detection of mitochondrial membrane potential; **b** cells were lysed, centrifuged and the supernatant mixed with luciferase/luciferin reagent, after which free ATP was detected with a luminometer. Data are expressed as the mean ± SD of triplicate experiments. Bars sharing a common letter are not significantly different at *p* < 0.05

In order to meet the cellular energy and processing demands during lipid metabolism, normal adipogenesis is accompanied by an increase in mitochondrial biogenesis [34]. At 48 h post inducement, we tested standard measures of mitochondria activity: reactive oxygen species (ROS) levels; mitochondrial membrane potential (MMP); and ATP production. The results show that relative to the control, the purpurin treatment resulted in decreased levels of ROS generation with the 100 μM, but not with the lower 50 μM dose. However, both mitochondrial membrane potential and ATP production were sharply curtailed with the lower 50 μM dose, with an additional small drop with the higher 100 μM dose (Fig. 4). These results show that adipogenesis-related mitochondrial biogenesis and/or activity was suppressed by the purpurin.

#### Expression and activation of proteins during differentiation

**Fig. 5:**
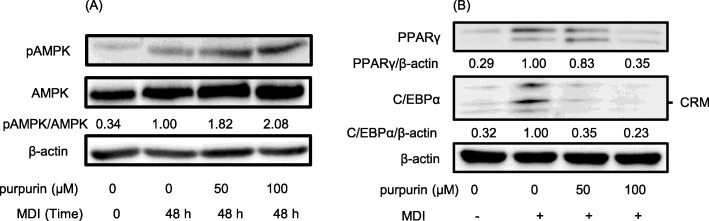
Western Blot analysis of intracellular proteins from purpurin-treated cells in: **a** early-stage differentiation (2 d after MDI inducement), and **b** late-stage differentiation (8 d after MDI inducement). Cells were treated for the first 48 h with MDI and purpurin, after which these substances were removed from the culture. Figures represent results from at least three individual experiments. AMPK activity was expressed as the ratio phosphorylated to non-phosphorylated AMPK. Protein expression was calculated relative to β-actin, a constitutively expressed cellular protein

The activation level of AMPK and the expression of PPARγ and C/EBPα, critical proteins related to the differentiation of adipocyte, were verified through Western blotting analysis. The results showed that the activation of AMPK (production of phosphor-form) was increased by purpurin treatment (Fig. 5a), and the level of expression of PPARγ and C/EBPα was decreased by purpurin treatment in a dose-dependent manner (Fig. 5b).

#### Lipid accumulation in fully differentiated cells

**Fig. 6:**
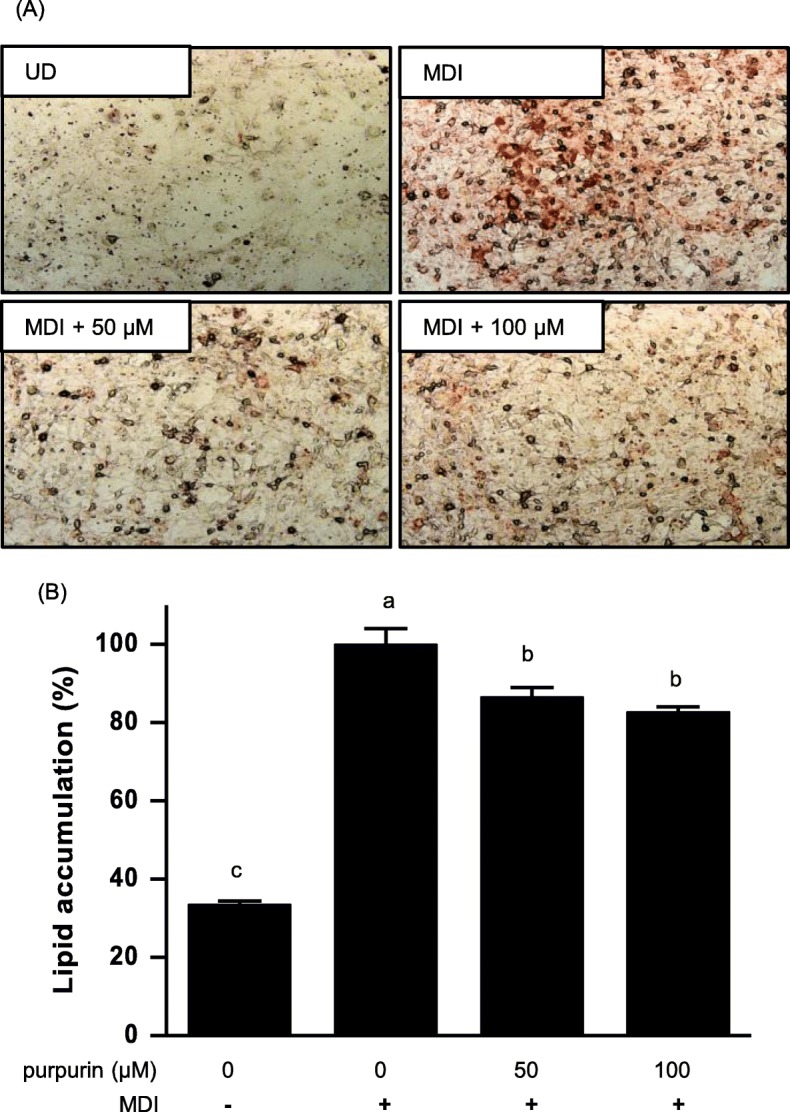
Effect of purpurin on lipid accumulation in differentiated 3T3-L1 cells as measured with Oil Red O staining. The accumulation of lipid was evaluated: **a** microscopically; and (**b**) colorimetrically (absorbance at 520 nm) from solubilized fat droplets collected from the stained cells. Data are expressed as the mean ± SD of triplicate experiments. Bars sharing a common letter are not significantly different at *p* < 0.05

At day 10 post-MDI administration, the control preadipocytes were fully differentiated to adipocytes. Lipid accumulation in the adipocytes was measured by Oil Red O staining. Microscopic examination of cells stained with Oil Red O showed that lipid accumulation was inhibited by purpurin in a dose-dependent manner (Fig. 6a). The results were quantified by measuring the absorbance of the solubilized stained lipid droplets at 520 nm. The results showed (Fig. 6b) that treatment with purpurin at 50 μM and 100 μM resulted in significant reductions of lipid accumulation in the cells (13 and 17%, respectively).

#### Triglyceride deposition in adipocytes

**Fig. 7:**
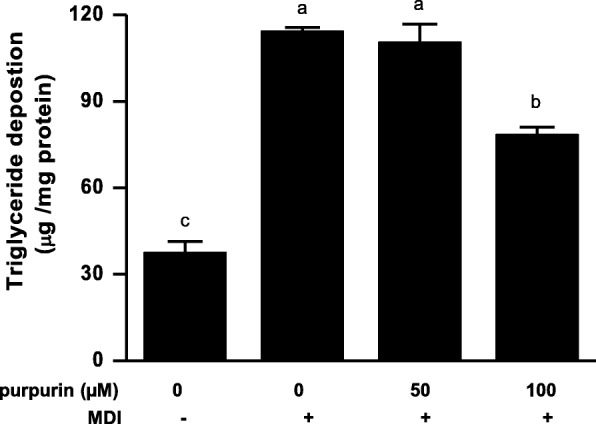
Effect of purpurin on triglyceride deposition in fully differentiated 3T3-L1 cells. Data are expressed as the mean ± SD of triplicate experiments. Bars sharing a common letter are not significantly different at *p* < 0.05

The inhibitory effect of purpurin on lipid accumulation was determined by measuring the triglyceride content of the differentiated adipocytes relative to their protein content. The result showed that there was no significant effect on triglyceride content for the 50 μM purpurin treatment, but triglyceride deposition was inhibited by 31% with the 100 μM purpurin concentration (Fig. 7).

### Effects of purpurin on mice consuming a high-fat diet

Obesity was induced in mice by feeding a high-fat diet (60% fat), and the effect of purpurin was investigated by adding 40 mg/kg and 80 mg/kg of purpurin to the diet.

#### Mouse body weights

**Fig. 8:**
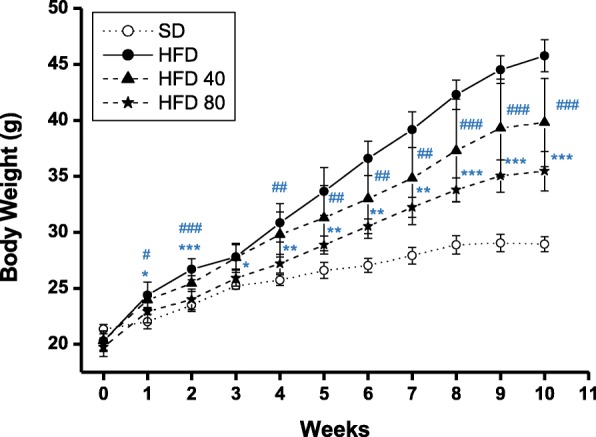
Body weight changes of male C57BL/6 mice fed a high-fat diet only (HFD), 40 mg/kg purpurin-contained high fat diet (HFD 40), or 80 mg/kg purpurin-contained high fat diet (HFD 80) for 10 weeks. # *p* < 0.5, ## *p* < 0.01, and ### *p* < 0.001 on means of HFD 40 versus high-fat diet. * *p* < 0.5, ** *p* < 0.01, and *** *p* < 0.001 on means of HFD 80 versus high-fat diet

The effect of purpurin on weight gain in mice fed a high-fat diet (HFD) was evaluated. The weight gain of mice fed the HFD was about 3.4-fold higher than that of the normal control diet group at 10 weeks. Oral administration of purpurin at the 40 mg/kg and 80 mg/kg level to the HFD resulted in reduced weight gain by 34 and 55%, respectively (Fig. 8, Table 1). In addition, the increased food efficiency ratio (FER) due to the high-fat diet, a 4-fold increase, was inhibited 32 and 45% by purpurin administration at dose of 40 mg/kg and 80 mg/kg, respectively, indicating that purpurin has the capacity to suppress weight gain due to ingestion of high fat level in a dose-dependent manner (Table 1).

**Table 1:** Effect of purpurin on weight gain, food uptake, and food efficiency ratio

	Standard diet (SD)	High-fat diet (HFD)	HFD, 40 mg/kg purpurin	HFD, 80 mg/kg purpurin
Weight gain (g/mouse)	7.59	25.49	19.49	15.72
Food uptake (g/day mouse)	3.00 ± 0.17^a^	2.47 ± 0.19^b^	2.49 ± 0.17^b^	2.32 ± 0.16^c^
Food efficiency ratio	3.61	14.72	11.19	9.69

Data are expressed as the mean ± SD of 10 mice experiments. Values sharing a common letter are not significantly different at *p* < 0.05

#### Glucose tolerance

**Fig. 9:**
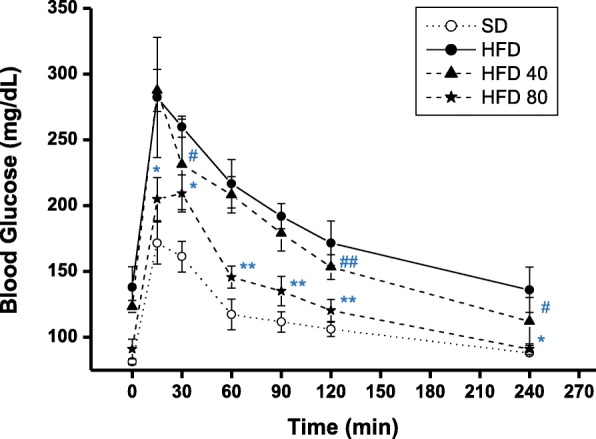
Effect of purpurin on glucose tolerance in high-fat diet fed mice. # *p* < 0.5, ## *p* < 0.01 on means of HFD 40 versus high-fat diet. * *p* < 0.5, ** *p* < 0.01 on means of HFD 80 versus high-fat diet

To test their response to a glucose challenge, the mice were fasted for more than 20 h, then treated orally with 1 mg/kg of glucose. Blood glucose was measured every 30 min after oral administration. The glucose challenge time-response curves show the purpurin treated mice had intermediate responses relative to the HFD and standard diets (Fig. 9). Moreover, blood glucose levels for the 80 mg/kg purpurin group, measured at 15, 30, and 120 min post-glucose administration, showed significant differences from HFD mice.

#### Plasma glucose and lipid levels

Blood samples taken at the time of animal sacrifice were evaluated for glucose, cholesterol, and triglycerides. The values for the parameters in the treatment groups fell between the positive and negative control except for triglycerides in the HFD 80 group, which fell below the standard diet group (Table 2). The HFD caused 173, 142, and 144% increases in blood glucose, cholesterol, and triglycerides levels, respectively. The 40 and 80 mg/kg purpurin treatments reduced blood glucose increases by 24 and 78%, cholesterol increases by 27 and 76%, and triglyceride increases by 64 and 123%, respectively.

**Table 2:** Effects of purpurin on plasma lipid profiles in high-fat fed mice

	SD	HFD	HFD 40	HFD 80
Glucose (mg/dL)	85.2 ± 7.9^b^	147 ± 18^a^	132 ± 17^a^	99 ± 13^b^
Total cholesterol (mg/dL)	107.0 ± 1.3^d^	151.6 ± 4.8^a^	139.7 ± 1.3^b^	117.49 ± 0.50^c^
Triglyceride (mg/dL)	85.2 ± 2.7^c^	123 ± 14^a^	98.7 ± 8.8^b^	76.4 ± 2.0^c^

Data are expressed as the mean ± SD of 10 mice experiments. Values sharing a common letter are not significantly different at *p* < 0.05.

#### Effect of purpurin on organ weights

Table 3 shows that purpurin did not seem to significantly increase or decrease the weights of the heart, lung, and kidney mouse organs, showing that although exposure of the mice to dietary purpurin for 10 weeks induced reduced weight gain of the mice, the anti-obesity effect did not result in the corresponding reduction in weights of individual mouse organs. These results might imply a lack of organ toxicity.

**Table 3:** Effect of purpurin on organ weights

	Organ weight (g)		
	Heart	Lung	Kidney
Normal diet	0.152 ± 0.026	0.171 ± 0.015	0.351 ± 0.035
High-fat diet only (HFD)	0.161 ± 0.015	0.180 ± 0.019	0.400 ± 0.019
HFD + 40 mg/kg purpurin	0.154 ± 0.017	0.183 ± 0.029	0.382 ± 0.039
HFD + 80 mg/kg purpurin	0.146 ± 0.029	0.178 ± 0.021	0.376 ± 0.038

Data are expressed as the mean ± SD of 10 mice experiments. No significant differences between test groups at *p* < 0.05

#### Hepatic lipid accumulation and liver injury

Livers were examined for weight changes, cholesterol and triglyceride levels, and for expression of hepatic enzymes (Table 4). In mice consuming the high-fat diet the liver weights increased 1.5-fold. Administration of purpurin at 40 and 80 mg/kg inhibited this increase by 65 and 96%, respectively. The triglyceride levels of the livers were also reduced by the dietary purpurin in a dose-dependent manner. Both doses of purpurin reduced the HFD-induced cholesterol gain to baseline levels. To assess for possible liver damage induced by a fatty liver, expression of the liver enzymes glutamate oxaloacetate transaminase (GOT) and glutamate pyruvate transaminase (GPT) were measured. GOT and GPT increased 13- and 7-fold, respectively in the HFD animals. The increase in GOT was reduced by 79 and 86%, and for GPT by 73 and 81%, for the 40 and 80 mg/kg doses, respectively. These results demonstrate that dietary purpurin protected the liver against the adverse effects of a high-fat diet.

**Table 4:** Effect of purpurin on the liver

	SD	HFD	HFD 40	HFD 80
Liver weight (g)	1.05 ± 0.08^c^	1.57 ± 0.08^a^	1.23 ± 0.15^b^	1.07 ± 0.02^c^
Triglyceride (mg/liver)	20.8 ± 1.9^d^	69.4 ± 5.0^a^	36.5 ± 3.5^b^	28.16 ± 0.75^c^
Total cholesterol (mg/liver)	2.86 ± 0.18^b^	7.25 ± 0.93^a^	3.80 ± 0.42^b^	3.00 ± 0.11^b^
GOT (IU/mL)	6.7 ± 1.3^c^	87.9 ± 4.9^a^	23.9 ± 3.8^b^	18.4 ± 2.2^b^
GPT (IU/mL)	11.7 ± 2.0^c^	85.5 ± 2.8^a^	31.3 ± 2.6^b^	25.8 ± 2.1^b^

Data are expressed as the mean ± SD of 10 mice experiments. Values sharing a common letter are not significantly different at *p* < 0.05

#### White adipose tissue

Changes in white adipose tissue resulting from the treatment with purpurin were evaluated (Table 5). HFD caused an increase in white adipose tissue weight (WAT), triglyceride levels, and adipose cell diameters by 5.0-, 10.5-, and 2.7-fold, respectively. The administration of 40 mg/kg purpurin in the diet had no effect on WAT weight, but reduced HFD-induced increases of triglycerides by 13%, and adipose cell diameters by 31%. Eighty milligram per kiliogram purpurin in the diet reduced these increases by 38, 59, and 71%, respectively.

**Table 5:** Effect of purpurin on white adipose tissue (WAT) weight, morphometry, and triglyceride contents

	SD	HFD	HFD 40	HFD 80
WAT weight (g)	0.40 ± 0.07^c^	2.03 ± 0.21^a^	2.00 ± 0.54^a^	1.41 ± 0.46^b^
Triglyceride (mg/WAT)	5.17 ± 0.21^d^	54.3 ± 1.0^a^	47.9 ± 1.2^b^	25.34 ± 0.28^c^
Diameter of adipose cells (μm)	247 ± 47^d^	654 ± 17^a^	528 ± 35^b^	364 ± 36^c^

Data are expressed as the mean ± SD of 10 mice experiments. Values sharing a common letter are not significantly different at *p* < 0.05

#### Histological change of liver and white adipose tissue

**Fig. 10:**
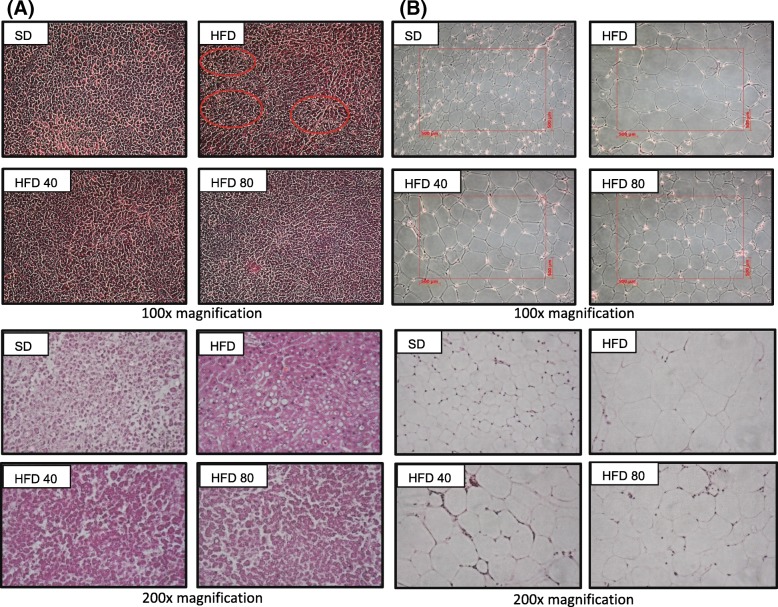
Histological changes of liver (**a**) and white adipose tissue (**b**) in mice fed a high-fat diet or a purpurin-containing high-fat diet. Each tissue was fixed with 4% paraformaldehyde, and sections were stained with hematoxylin and eosin Y (H&E). Figures represent results from at least three separate experiments

Histological changes in liver and white adipose tissue were also evaluated. The administration of purpurin at 40 mg/kg or 80 mg/kg resulted in the reduction of accumulated liver fat (Fig. 10a, red circle), as well as in the size of white adipose tissue cells (Fig. 10b, red square). The HFD caused the adipose tissue cell diameters to more than double, relative to the control. Approximate diameters were 300 μm for control mice (SD), 800 μm for mice on the HFD, 600 μm for mice on the HFD40, and 500 μm for mice on the HFD80 diets. These observations, taken together with the chemistry and biochemistry assays, indicate that purpurin decreased the hypertrophy (fat storage), as well as the hyperplasia (number) of the adipose cells.

## Discussion

The animal studies showed that purpurin mitigated the negative effects of a HFD as evidenced by: (a) reduced weight gain; (b) increased glucose tolerance; (c) reduced plasma glucose, cholesterol, and triglycerides; (d) reduced fatty liver indicators, including liver weight, lipid and cholesterol accumulation, and liver enzymes (GOT and GPT); and (e) reduced white adipose tissue weight, adipose cell size, and adipose lipid accumulation. The food intake was unchanged for the 40 mg/kg purpurin group but decreased slightly (6%) for the 80 mg/kg purpurin group, which could account for some of the changed indicators. Other organ weights were not affected by purpurin consumption, indicating non-toxicity.

The 3T3-L1 cell cytotoxicity assay showed that purpurin was not toxic at the concentrations used in this study for the cell assays: 50 and 100 μM. Zengin, Degirmenci, Alpsoy and Aktumsek [2] found neither purpurin nor alizarin was cytotoxic to L926 fibroblast cells. Ino, Tanaka, Okumura, Morishita, Makita, Mori, Kato and Nakamura [5] estimated the maximum tolerated oral dose in mice of the whole plant *Rubia tinctorum*, containing both anthraquinones purpurin and alizarin in glycosylated form, to be 3.5–5 g/kg body weight. With *Rubia tinctorum* containing about 1% alizarin [35], this corresponds to about 35–50 mg/kg alizarin plus a smaller amount of purpurin; which is similar to the doses chosen in our oral feeding study of 40 and 80 mg/kg body weight purpurin. The Ino, Tanaka, Okumura, Morishita, Makita, Mori, Kato and Nakamura [5] study found no clinical signs of toxicity nor weight loss in mice fed up to 5% of their diet as *Rubia tinctorum* for 90 days. The lack of weight loss in their feeding study suggests that the weight loss in our study is related only to HFD-induced weight gain, and not to a failure to thrive.

Cell adipogenesis was apparently inhibited by purpurin by way of activation of AMPK (Fig. 5), an enzyme that modulates cellular energy homeostasis [36]. Purpurin treatment of 3T3-L1 cells at the 100 μM level doubled the activation level of AMPK relative to the control at 48 h. There are many possible drivers for activation of AMPK, but the most common is a high AMP to ATP ratio [37]. AMPK is activated during a low energy states by sensing falling ATP levels, specifically high AMP/ATP ratios within the cell [37]. The low ATP levels in the treated cells (Fig. 4) suggests that the purpurin mechanism is an AMP-dependent one [38]. A cellular energy regulator, pAMPK drives catabolic and minimizes anabolic processes in the cell as a means to return the cell to homeostasis [37]. Among other targets, pAMPK inhibits the transcription factors C/EBPα and PPARγ [39], which are necessary for adipogenesis. Consistent with this dependence, we measured a decrease in C/EBPα and PPARγ transcription in the 3T3-L1 cells, leading ultimately to a decrease in adipogenesis as evidenced by the fat accumulation in the cells at day 10 post-inducement.

Analysis of adipogenesis-induced changes in 3T3-L1 cells showed that purpurin sharply curtailed the increased mitochondrial activity/biogenesis normally observed during early-stage differentiation. Mitochondrial membrane potential, ATP production, and ROS generation were all lower in the 100 μM purpurin-treated cells relative to the untreated cells (Fig. 4), but only the first 2 measures were lowered in the 50 μM purpurin treatment. Because of ROS changes lagged behind the other measures, we hypothesize that ROS was reduced as a result of decreased mitochondrial activity. Increased mitochondrial biogenesis, including generation ROS and ATP production, is essential to the initiation of adipogenesis [34, 40]. It remains to be determined how purpurin acts on the mitochondria.

## Conclusions

The investigation of the bioactivity of the anthraquinone purpurin has shown that purpurin has in vivo anti-obesity and in vitro anti-adipogenic effects. Mice on a high-fat diet with added purpurin experienced a 55% reduction in weight gain compared to those on the HFD control diet without added purpurin, and the effect was dose-dependent. In vitro, differentiation of 3T3-L1 cells was dose-dependently inhibited by purpurin, apparently by the activation of AMPK. It appears likely that AMPK activation was due to diminished ATP production from reduced mitochondrial biogenesis. Additional studies might elucidate the mechanism of the effect of purpurin on the mitochondria and determine the in vivo antioxidative capacity, as well as confirm the safety of the compound.

## Data Availability

The datasets used and/or analyzed during the current study are available from the corresponding author on reasonable request.

## Abbreviations

AMPK: Adenosine 5′-monophosphate-activated protein kinase
C/EBPα: CCAAT enhancer binding protein α
HFD: High-fat diet
MDI: MIX-DEX-Insulin differentiation medium
MMP: Mitochondrial membrane potential
PPARγ: Peroxisome proliferator activated receptor-γ
ROS: Reactive oxygen species
